# Metabarcoding insights into the fungal diversity and biotechnological potential of mangrove sediments in Ecuador’s Reserva Ecológica Manglares Churute

**DOI:** 10.3389/ffunb.2026.1710970

**Published:** 2026-02-10

**Authors:** Xavier Álvarez-Montero, Ingrid Mercado-Reyes, Wilian Castillo-Chamba, Geovanny Gordillo, Efrén Santos-Ordóñez, Diego Portalanza, Angélica Saeteros-Hernández

**Affiliations:** 1 Departamento de Ciencias de la Vida y Agricultura, Universidad de las Fuerzas Armadas-ESPE, Santo Domingo, Ecuador; 2 Laboratorio de Inocuidad Alimentaria, Escuela de Medicina Veterinaria, Universidad Nacional Andrés Bello (UNAB), Santiago, Chile; 3 Doctorado en Biotecnología, Facultad de Ciencias de la Vida, República, Universidad Nacional Andrés Bello (UNAB), Santiago, Chile; 4 Facultad de Medicina Veterinaria, Universidad Agraria del Ecuador, Guayaquil, Ecuador; 5 Pontificia Universidad Católica del Ecuador, Quito, Ecuador; 6 Universidad Pública de Santo Domingo de los Tsáchilas - UPSDT, Quevedo, Ecuador; 7 Centro de Investigaciones Biotecnológicas del Ecuador, ESPOL Polytechnic University, ESPOL, Guayaquil, Ecuador; 8 Facultad de Ciencias de la Vida, ESPOL Polytechnic University, ESPOL, Guayaquil, Ecuador; 9 Facultad de Ciencias Agrarias, Instituto de Investigación, Universidad Agraria del Ecuador, Guayaquil, Ecuador; 10 Departamento de Física, Universidade Federal de Santa Maria, Santa Maria, Brazil; 11 Facultad de Salud Pública, Escuela Superior Politécnica del Chimborazo (ESPOCH), Riobamba, Ecuador

**Keywords:** Ascomycota, biodiversity, Ecuador, ITS sequencing, mangrove fungi, metabarcoding

## Abstract

Mangrove ecosystems are biodiversity hotspots and vital carbon sinks, yet their fungal communities—key drivers of nutrient cycling and ecosystem resilience—remain largely unexplored in the Neotropics. This is particularly true for Ecuador's protected reserves, where no molecular census of sediment fungi exists. To address this gap, we conducted the first metabarcoding survey of the fungal microbiome in the sediments of the Reserva Ecológica Manglares Churute (REMC), a critical mangrove habitat under increasing anthropogenic pressure. This is the first molecular study to characterize fungal communities in the mangrove sediments of the Reserva Ecológica Manglares Churute (REMC) in the neotropical context of Ecuador, using metabarcoding. The fungal community was dominated by Ascomycota (68%) and Basidiomycota (30%), with minor contributions from Mortierellomycota, Chytridiomycota, and Mucoromycota (<0.1%), and 2.10% unclassified at the phylum level. The most diverse sample (2005L2-69) had a Shannon index of 2.166. Rarefaction curves indicated that additional sampling could reveal more fungal diversity. Fungal assemblages were similar across samples, with minor variations linked to environmental factors. Predominant classes included Dothideomycetes, Agaricomycetes, and Eurotiomycetes. At the genus level, *Ascochyta* (27%), *Antrodia* (24%), and *Talaromyces* (17%) were the most abundant. The presence of genera such as *Talaromyces* and *Penicillium* highlights their biotechnological potential, like antibacterial properties. At the same time, the abundance of *Ascochyta*, a phytopathogen, suggests potential stress or disease susceptibility in this area of REMC. This research provides an essential preliminary overview of the mycobiome in this underexplored region and identifies principal taxa of ecological and biotechnological importance. Furthermore, this investigation, conducted in a specific area of REMC sediments, employs metabarcoding analysis utilizing ITS86-ITS4 primers in Neotropical mangroves, thereby contributing to the global understanding of mangrove microbiomes.

## 1 Introduction

Mangroves are distinctive, highly productive ecosystems that sustain diverse biological communities. These environments are recognized for their role in safeguarding coastlines through pollutant filtration, erosion prevention, buffering against natural disasters, and providing nursery habitats for numerous marine species, thereby supporting fisheries (Holguin et al., 2001; Thatoi et al., 2013; Allard et al., 2020; Wainwright et al., 2023). Overall, mangroves possess substantial ecological importance due to the intricate biological processes that render these ecosystems highly productive, influenced by factors such as salinity fluctuations, nutrient availability, and the abundance of organic matter and carbon (Thatoi et al., 2013; Wainwright et al., 2023).

Numerous processes are regulated by the mangrove microbiota, which encompasses diverse groups of microorganisms inhabiting marine, freshwater, soil, and rhizosphere niches. The mangrove microbiota plays a crucial role in carbon flux, nitrogen fixation, phosphate solubilization, and the decomposition of recalcitrant organic matter such as cellulose and lignin (Wainwright et al., 2023). Accordingly, the microbial community within this environment is indispensable for maintaining productivity, nutrient supply, and the preservation of mangrove vegetation (Kundu et al., 2024).

Generally, these microorganisms comprise bacteria and fungi (91%), algae (7%), and protozoa (2%) (Palit et al., 2022). Given that mangroves are ecosystems grounded in detritus, fungi assume a vital role as decomposers of resilient organic matter, thereby supporting the nutrient cycle within the ecosystem. According to Kundu et al., the diversity of fungi in mangrove ecosystems is contingent on factors such as mangrove age, plant species diversity, and physical-chemical parameters, including temperature, salinity, and tidal range (Kundu et al., 2024).

The investigation of fungal diversity using traditional, morphology-based methodologies is inherently limited, as many taxa either do not readily sporulate in culture or cannot be cultivated under laboratory conditions. This substantial portion of the microbial community, often referred to as microbial “dark matter” (Li S. et al., 2023), remains obscured by specific nutritional requirements or complex ecological interactions. To overcome these limitations, molecular techniques such as DNA metabarcoding have become essential for acquiring a more comprehensive understanding of fungal biodiversity (Cuadros-Orellana et al., 2013; Nam et al., 2015; Chi et al., 2019). The effectiveness of this approach is demonstrated in mangrove ecosystems. For example, in sediments from China’s Zhanjiang National Mangrove Reserve, ITS-based metabarcoding identified 125 species across 64 genera—an extent of diversity unlikely to be fully documented through cultivation alone (Li M. et al., 2023). Similarly, a study of Singaporean mangroves utilized metabarcoding to identify Eurotiomycetes and *Trichoderma* as the dominant taxa in surface and subsurface sediments, respectively, showcasing the method’s ability to elucidate ecological patterns across sediment layers.

Metabarcoding has elucidated consistent patterns within mangrove fungal communities across principal biogeographic regions, notwithstanding notable geographical and methodological gaps. In Asian mangrove ecosystems, investigations using the ITS1/ITS2 regions have characterized a core community composed primarily of Ascomycota and Basidiomycota, with Dothideomycetes and Eurotiomycetes as the predominant classes inhabiting leaves and sediments (Chi et al., 2019; Lee et al., 2019; Pothayi et al., 2024). The analogous dominance of these phyla has been affirmed by the limited number of Neotropical studies conducted in Brazil and Puerto Rico (Urbina et al., 2016; Calvacante et al., 2019; Martins de Oliveira et al., 2025), indicating a potentially universal functional framework for mangrove mycobiomes.

Nevertheless, this foundational knowledge exhibits significant spatial and methodological disparities. Primarily, research efforts are predominantly focused on Asian coastlines, thereby leaving the fungal diversity within Pacific South American mangroves—a distinct biogeographic and climatic region—virtually unexplored at the molecular level. Secondly, within the Neotropics, existing studies have either focused on specific substrates (e.g., lichenized fungi) (Calvacante et al., 2019) or on wetlands adjacent to mangroves (Portalanza et al., 2025). As a result, there is an absence of a comprehensive, DNA-based profile of the sediment fungal community within a protected Ecuadorian mangrove reserve. This deficiency impedes our capacity to understand regional microbial biodiversity, evaluate ecosystem health, and determine the conservation significance of these vital habitats.

In Ecuador, mangroves are distributed along the coastal profile of the provinces of Esmeraldas, Manabí, Guayas, and El Oro, including the Galápagos Islands. The most significant areas of these ecosystems are found in the estuaries of the Mataje-Santiago-Cayapas, Muisne, Cojimíes, Chone, Guayas, and Jubones-Santa Rosa-Arenillas rivers, with the Guayas River estuary and the Gulf of Guayaquil possessing the largest surface areas (Cornejo, 2014). Mangroves deliver vital ecosystem services that sustain human communities, including the harvesting of aquatic species (López-Rodríguez, 2018), timber for construction, firewood, and medicinal plants (Bandaranayake, 1998). Furthermore, mangroves offer regulatory ecosystem services, such as protecting populations from natural disasters (McIvor et al., 2015). Nonetheless, in Ecuador, the mangrove ecosystem faces threats arising from the development and expansion of shrimp farms, industrial and urban infrastructure, pollution, deforestation, overexploitation of fisheries, and climate change (Le, 2023). Despite the wide range of ecosystem services mangrove forests provide, they are among the most threatened ecosystems globally. Several studies have reported a loss of approximately 135,882 km² between 2000 and 2016, mainly due to human activities (Hamilton and Casey, 2016; Morocho et al., 2022; Leal and Spalding, 2024). In Ecuador, as of 2018, the mangrove forest covered approximately 1,642.2 km². This extension results from several conservation plans aimed at mitigating the impacts of shrimp farming, which is the main threat (Morocho et al., 2022).

Given the pivotal role of fungi in mangrove ecosystem functioning and the paucity of molecular data from the Neotropics, the research focused on the Reserva Ecológica Manglares Churute (REMC) in coastal Ecuador. This “Strict Nature Reserve” (IUCN category) constitutes one of Ecuador’s largest and most significant protected mangrove ecosystems, recognized as a RAMSAR site since 1990. The investigation was conducted along the “La Flora” trail, an area subject to substantial anthropogenic pressure from tourism. Despite its high conservation status and ecological significance, the microbial diversity within REMC sediments has remained largely unexplored at a molecular level. Prior efforts have been confined to morphological descriptions of a limited subset of cultivable fungi (Álvarez et al., 2019; Tamayo-Cevallos and Álvarez-Montero, 2022), leaving the majority of the mycobiome—including unculturable taxa and community composition—uncharacterized. This study thus presents the inaugural metabarcoding evaluation of sediment fungal communities in the REMC, establishing a vital baseline for comprehending microbial diversity and ecological function within a protected yet potentially impacted Neotropical mangrove.

## 2 Materials and methods

### 2.1 Sample collections

**Figure 1:**
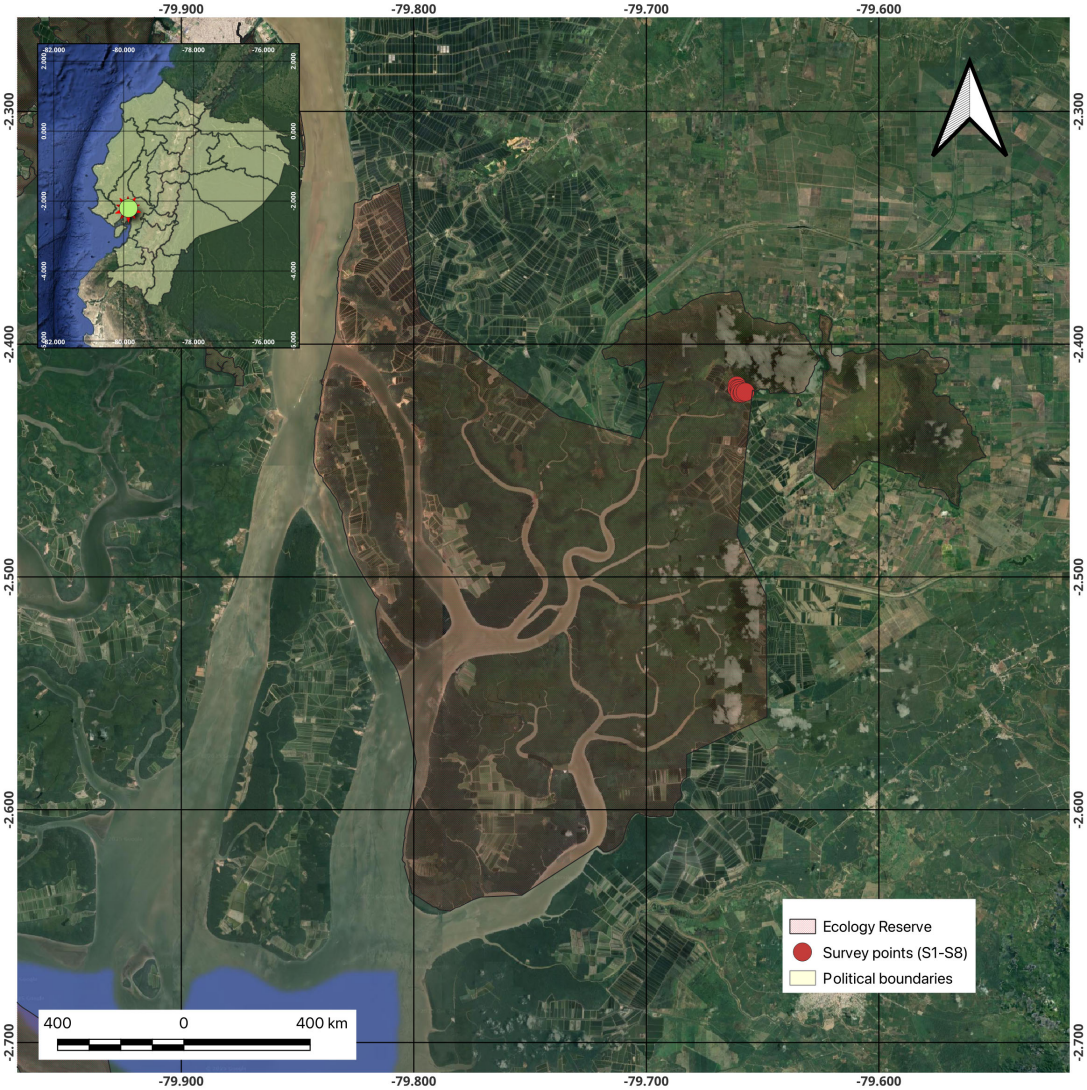
Location of the Reserva Ecológica Manglares Churute and sampling zone along the “La Flora” trail.

The study area corresponds to a delimited zone of the Reserva Ecológica Manglares Churute (REMC), located on the western edge of the estuary formed by the confluence of the Guayas River and the Gulf of Guayaquil. With an area of 493.89 km², the REMC is one of the largest marine-coastal reserves in Ecuador. The reserve encompasses mangrove forests, numerous estuaries and inlets, a freshwater lagoon known as El Canclón, and hills rising up to 680 meters above sea level, which form part of the Churute mountain range (**Figure 1**).

Sampling for this study was conducted along the “La Flora” trail within the REMC during the March 2021 rainy season. Eight fixed sampling stations were established, spaced approximately 100 meters apart (see **Table 1**). MAATE identified the precise location of the sampling site under research authorization no. 015-2018-IC-FLO/FAU-DPAG/MAE to minimize ecosystem disturbance, considering its status as an ecotourism trail. The “La Flora” trail was chosen as a representative example of anthropogenic impact (tourism pressure). The eight stations were positioned 100 meters apart to capture the spatial heterogeneity along this trail effectively. Although this design documented spatial variation along the trail, environmental parameters such as salinity, pH, and organic matter content were not measured. Future research should incorporate these metrics to establish a direct correlation between fungal community composition and environmental factors. At each station, ten randomly established 1 m² areas were sampled, and ten sediment samples—one per square meter—were collected at a depth of 10 cm during low tide. The samples were placed into sterile, hermetically sealed, pre-labeled plastic bags, stored in a thermal container at 4°C for preservation, and transported to the laboratory for subsequent analysis. Once in the laboratory, the samples were stored at -20°C to preserve DNA integrity until DNA extraction.

**Table 1:** Geographic coordinates of sampling stations in the Reserva Ecológica Manglares Churute in Degrees, Minutes, and Seconds (DMS) format.

Station	Sample code	Latitude	Longitude
1	2005L-68	2°25´04´´S	79°39´40´´W
2	2005L-69	2°25´09´´S	79°39´40´´W
3	2005L-70	2°25´09´´S	79°39´37´´W
4	2005L-71	2°25´12´´S	79°39´36´´W
5	2005L-72	2°25´16´´S	79°39´36´´W
6	2005L-73	2°25´16´´S	79°39´32´´W
7	2005L-74	2°25´14´´S	79°39´31´´W
8	2005L-75	2°25´14´´S	79°39´27´´W

### 2.2 DNA extraction, sample preparation, PCR conditions, and sequencing

After 48 h of collection, sediment samples from each station were homogenized, and then 0.25 g of environmental DNA was extracted from each homogenized sediment sample using the Soil DNA Isolation Plus kit (Norgen Biotek Corp., 3430 Schmon Parkway, Thorold, ON L2V 4Y6, Canada), following the manufacturer’s instructions (available at: https://norgenbiotek.com/sites/default/files/resources/PI64000-5%20Soil%20DNA%20Isolation%20Plus%20Kit%20Insert.pdf).

For the metabarcoding analysis, a minimum of 1 μg of DNA per sample was used for sequencing on the Illumina MiSeq platform. Library preparation and sequencing were performed commercially using ITS primers (www.biosequenceec.com), combining three biological replicates per station in equimolar concentrations.

The sequencing procedure followed the Fungal Metagenomic Sequencing Demonstrated Protocol (Illumina, San Diego, CA, USA), adapted for metabarcoding by amplifying the ITS86–ITS4 region of the fungal rRNA gene cluster (Waud et al., 2014; Garces-Fiallos et al., 2022). Briefly, amplicons were generated, prepared for sequencing following standard library preparation steps, and sequenced on the Illumina MiSeq System with 2 × 301 bp paired-end reads. Raw data were stored in FASTQ format and uploaded to the Illumina BaseSpace Sequencing Hub for further analysis.

### 2.3 Bioinformatic analysis of metabarcoding data

The sequencing libraries were analyzed using the ITS Metagenomics App available on the Illumina BaseSpace Sequence Hub (www.basespace.illumina.com/dashboard). Raw reads from each sample were processed following the methodology described by Garces-Fiallos et al., using the UNITE Fungal ITS Database v7.2 as the reference for taxonomic classification (Garces-Fiallos et al., 2022). Amplicons corresponding to the ITS86–ITS4 region, located within the Internal Transcribed Spacer 2 (ITS2) of the fungal rRNA gene cluster, were used for the identification of fungal taxa (Embong et al., 2008; Schoch et al., 2012).

Each read was aligned with the UNITE database reference sequences, and groups of reads matching a specific taxonomic level were clustered to generate count data. This count matrix was then used to determine the fungal community composition across various taxonomic ranks, including kingdom, phylum, class, order, family, genus, and species. Reads that could not be confidently assigned at a given taxonomic rank were retained and reported as ‘unclassified’ at that rank.

### 2.4 Biodiversity analysis

Alpha diversity was estimated using synecological indices, including Dominance, Shannon diversity, Richness, Pielou’s Evenness, and the Chao1 richness estimator. Let $n_{i}$ be the number of reads (or individuals) of the i-th taxon in a given sample, $N=\sum_{i=1}^{S}n_{i}$ the total number of reads in that sample, S the observed richness (number of taxa), and $p_{i}=n_{i}/N$ the relative abundance of taxon i.

Simpson’s Dominance index ( D) was computed as (Equation 1):

(1) $$D=\sum_{i=1}^{S}p_{i}^{2}$$

Shannon diversity ( $H^{\prime }$) was calculated as (Equation 2)

(2) $$H^{\prime }=-\sum_{i=1}^{S}p_{i}\text{ ln }p_{i}$$

Observed richness ( S) corresponds to the total number of taxa detected in each sample (Equation 3):

(3) $$S=\sum_{i=1}^{S}1$$

Pielou’s Evenness ( $J^{\prime }$) was calculated as the ratio between the observed Shannon diversity and its maximum possible value for a given richness (Equation 4):

(4) $$J^{\prime }=\frac{H^{\prime }}{\ln S}$$

The Chao1 estimator ( ${\hat{S}}_{Chao1}$) was used as a non-parametric estimator of true species richness (Equation 5):

(5) $${\hat{S}}_{Chao1}=S_{\text{obs}}+\frac{F_{1}^{2}}{2F_{2}}$$

where $S_{\text{obs}}$ is the observed richness, $F_{1}$ is the number of taxa represented by a single read (singletons), and $F_{2}$ is the number of taxa represented by exactly two reads (doubletons).

To evaluate beta diversity and differences in fungal assemblages among samples, Bray–Curtis dissimilarities were calculated from the taxon-by-sample abundance matrix. For two samples i and j, with abundances $x_{ik}$ and $x_{jk}$ of taxon k, the Bray–Curtis dissimilarity ( $d_{ij}$) was defined as (Equation 6):

(6) $$d_{ij}=\frac{\sum_{k=1}^{S}|x_{ik}-x_{jk}|}{\sum_{k=1}^{S}\left(x_{ik}+x_{jk}\right)}$$

Principal Coordinate Analysis (PCoA) was then applied to the Bray–Curtis distance matrix to represent multivariate differences in community composition in a reduced-dimensional space. Let $D=\left(d_{ij}\right)$ be the n×n matrix of pairwise dissimilarities among n samples. First, the squared dissimilarities were converted into a centered matrix (Equation 7):

(7) $$A_{ij}=-\frac{1}{2}d_{ij}^{2}$$

A double-centering operation was then applied (Equation 8):

(8) B=JAJ

where $J=I-\frac{1}{n}11^{⊤}$, I is the identity matrix, and 1 is a column vector of ones.

The matrix B was decomposed by eigenanalysis (Equation 9):

(9) $$B=Q\text{Λ}Q^{⊤}$$

where Q is the matrix of eigenvectors and Λ is the diagonal matrix of eigenvalues. The principal coordinates (PCoA axes) of each sample were obtained as (Equation 10):

(10) $$Y=Q{\text{Λ}}^{\mathrm{1/2}}$$

with the columns of  representing the ordination axes used in subsequent graphical representations.

Non-metric multidimensional scaling (NMDS) was also applied to the Bray–Curtis dissimilarity matrix to obtain an alternative, rank-based representation of community dissimilarities. NMDS solutions were evaluated using Kruskal’s Stress-1 as a measure of goodness of fit.

All alpha diversity indices and Bray–Curtis dissimilarities were computed using the BiodiversityR package in R (Kindt and Coe, 2005). Ordination analyses (PCoA and NMDS) [30] were performed with the vegan package.

Sample-based and read-based rarefaction/extrapolation curves were generated using the iNEXT package in R, using Hill number q = 0 (taxonomic richness). For the sample-based analysis, an incidence dataset (presence/absence of taxa across the eight stations) was used to estimate richness as a function of the number of samples. For the read-based analysis, abundance data (read counts per taxon; num_hits) were used to estimate richness as a function of sequencing depth for each station. In both cases, 95% confidence intervals were obtained by bootstrap resampling (bootstrap replications), and plots display interpolation (rarefaction) as solid lines and extrapolation as dashed lines (Chao et al., 2014).

To explore patterns of co-occurrence among fungal taxa, pairwise Spearman rank correlation coefficients (ρ) were calculated from their relative abundance profiles across the eight stations. The resulting correlation matrix was generated with the Hmisc package in R and visualized as a clustered heatmap with ggplot2, using hierarchical clustering to group taxa with similar correlation profiles. Strong positive correlations (ρ > 0.7) were interpreted as indicative of potential co-occurrence or shared niche preferences.

## 3 Results

A total of 1,027,731 reads were obtained and successfully assigned to specific taxa across all samples, with an average sequencing depth of approximately 128,466 reads per sample. Overall, sample 2005L2–68 yielded the highest number of taxonomically assigned reads (228,296), while sample 2005L2-71 had the lowest number of reads (18,338). Across all samples, more than 85% of reads were classified at the genus level. The most species-rich sample was 2005L2-70, with 574 species identified, whereas sample 2005L2-71 again showed the lowest species richness, with only 204 species.

### 3.1 Taxonomic composition

**Figure 2:**
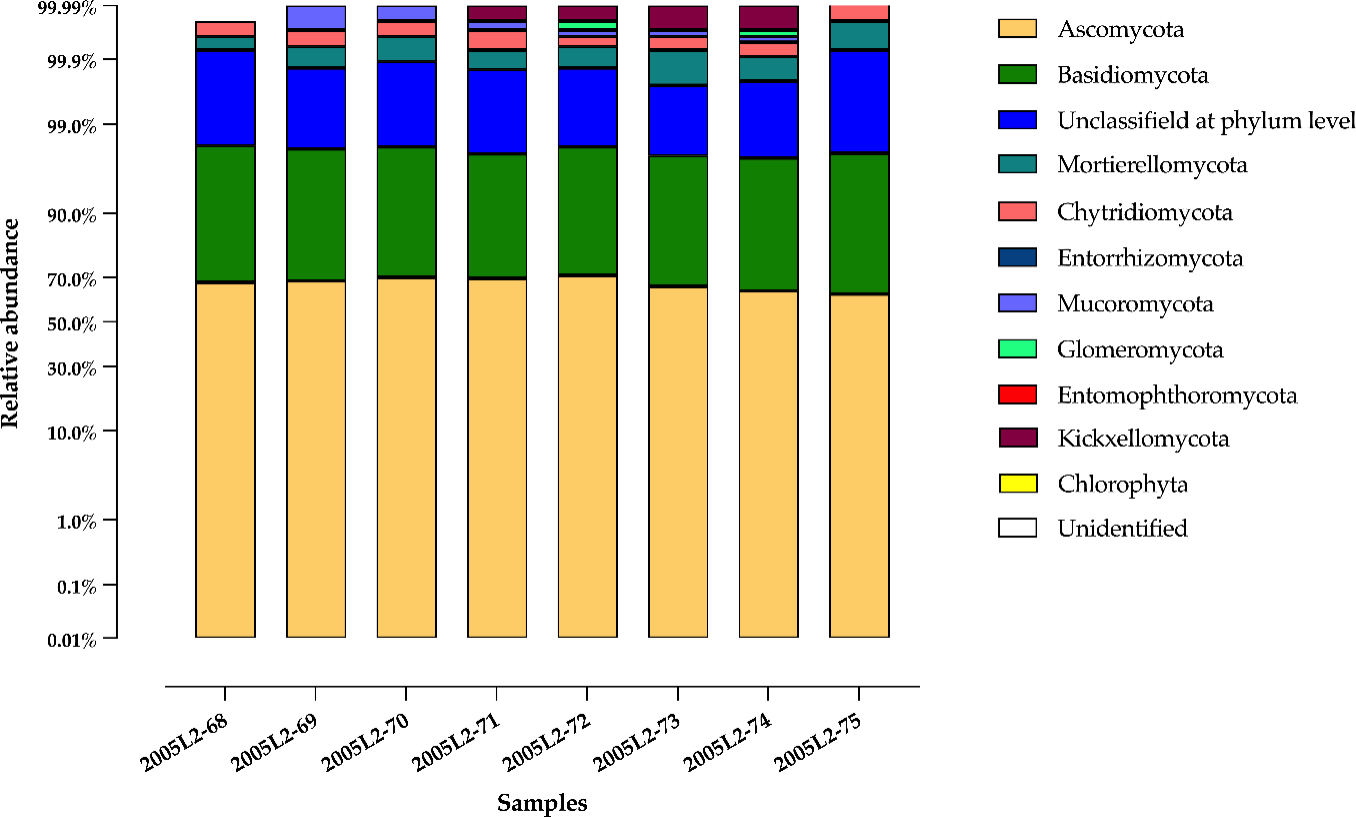
The taxonomic composition distribution of the more abundant phyla in the mangrove sediment of REMC.

A total of 10 fungal phyla were identified from the sediment samples. Phyla with low read counts (<50 reads) were grouped into an “Others” category. Among them, Ascomycota (with a relative abundance of 67.52 ± 2.84%) and Basidiomycota (with a relative abundance of 30.24 ± 2.65%) were the most abundant across all samples (**Figure 2**). Additionally, 2.10 ± 0.25% of the sequences remained unclassified at the phylum level, which may reflect the presence of poorly represented or novel taxa in the reference database. Other phyla, including Mortierellomycota, Chytridiomycota, Mucoromycota, Glomeromycota, Kickxellomycota, and the division of green algae Chlorophyta, appeared at very low relative abundances (<0.1%).

**Figure 3:**
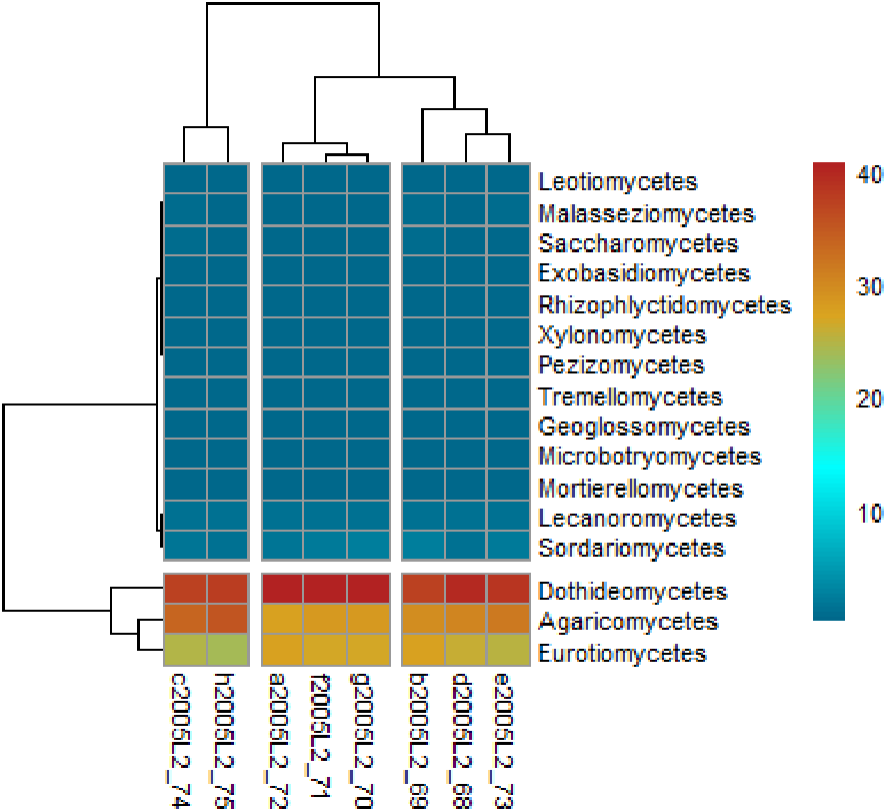
Heat map of the relative abundance of fungal classes on mangrove sediment samples.

Overall, 37 fungal classes were detected, with Dothideomycetes and Agaricomycetes being the most prevalent, followed by Eurotiomycetes, with 37.54 ± 1.49%, 29.42 ± 2.65%, and 25.00 ± 1.33%, respectively. However, class distribution varied between samples (**Figure 3**). Within the phylum Ascomycota, 21 classes were identified. The most abundant among these were Dothideomycetes, Eurotiomycetes, Sordariomycetes, Lecanoromycetes, and Saccharomycetes, with relative abundances of 37.5%, 25.0%, 1.6%, 1.1%, and 0.4%, respectively.

Similarly, within Basidiomycota, fourteen classes were identified, with Agaricomycetes (29.5%) being the most abundant. Other detected classes include Malasseziomycetes, Tremellomycetes, and Microbotryomycetes; however, each accounted for less than 1% of the total relative abundance.

At the genus level, the community was predominantly composed of a limited number of taxa, with six genera—*Ascochyta*, *Antrodia*, *Talaromyces*, *Penicillium*, *Ceramothyrium*, and *Rhizoctonia*—collectively representing more than 75% of the total relative abundance across all samples (refer to **Supplementary Table S1** for complete data).

**Figure 4:**
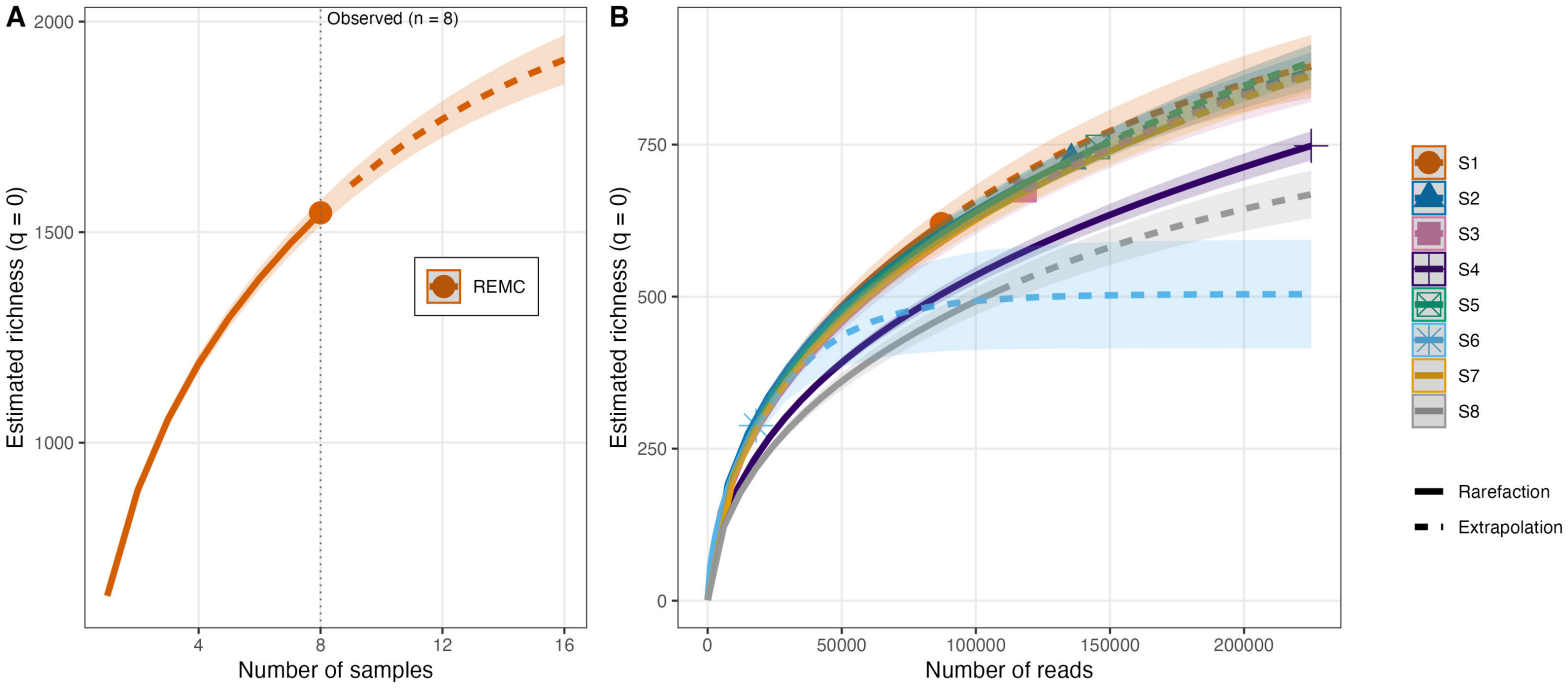
Rarefaction and extrapolation of fungal richness in mangrove sediment samples from the Reserva Ecológica Manglares Churute (REMC). **(A)** Sample-based rarefaction/extrapolation curve for incidence data (presence/absence across sampling units), showing estimated richness (Hill number q = 0) as a function of the number of samples. The solid segment represents interpolation (rarefaction) and the dashed segment extrapolation beyond the observed effort (n = 8 samples); shaded bands indicate 95% confidence intervals estimated from 50 bootstrap replications. **(B)** Read-based rarefaction/extrapolation curves (q = 0) for each station (S1–S8) based on abundance data (num_hits), showing richness as a function of sequencing depth (number of reads).

Furthermore, the rarefaction/extrapolation analyses indicate that sampling effort remains a limiting factor for recovering total fungal richness in this study area (**Figure 4A**). The sample-based curve does not reach an asymptote at the observed effort ( n=eightstations), and extrapolation suggests that additional sampling would likely yield further taxa. In contrast, the read-based curves by station (**Figure 4B**) show diminishing returns in estimated richness with increasing sequencing depth for most samples, supporting that per-sample sequencing depth was generally sufficient to characterize within-sample diversity. Together, these results indicate that additional stations (rather than additional reads per station) are the primary path to capturing a larger fraction of the REMC sediment mycobiome.

Regarding species-level composition in the mangrove sediment samples from the Churute Mangroves Ecological Reserve, the most abundant species identified were *Didymella rabiei* (teleomorph) (*Ascochyta rabiei* anamorph) (26.93 ± 1.30), *Antrodia* sp. (21.84 ± 2.41), and *Talaromyces ruber* (15.15 ± 0.64). These were followed by less abundant species, including *Penicillium corylophilum (*4.91 ± 0.36*)*, *Didymellaceae* sp. *(*1.54 ± 0.11*)*, and *Ceramothyrium melastoma (*1.41 ± 0.11*)*. At the same time, 11.10 ± 0.93 % was grouped under “Other,” which comprises all species with a relative abundance below 3.50 %, excluding those previously mentioned. Across all fungal reads, 16.7% remained unclassified at the genus level and 40.8% at the species level.

**Figure 5:**
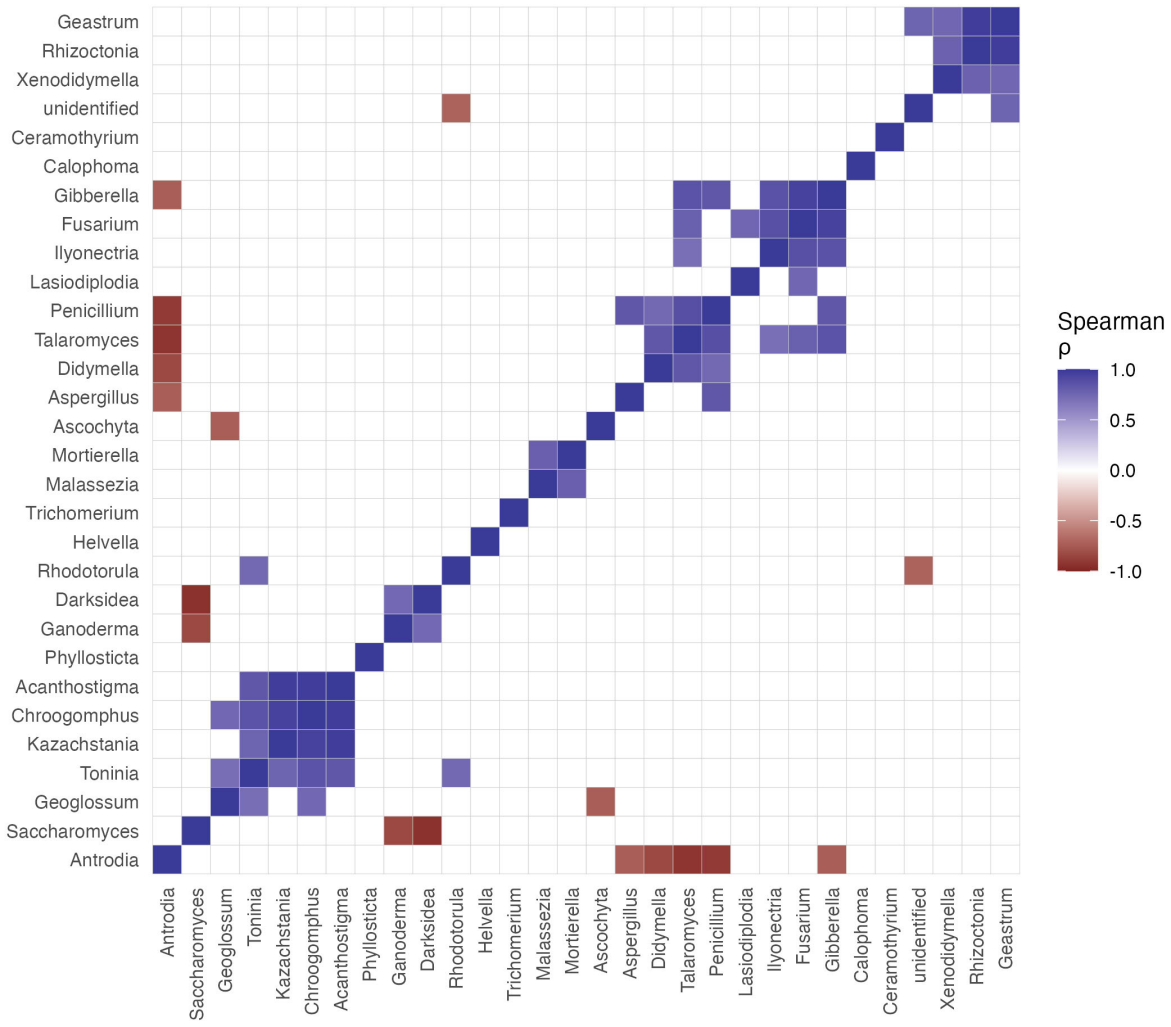
Co-occurrence patterns among dominant fungal genera in mangrove sediments of REMC. Heatmap showing pairwise Spearman rank correlation coefficients (ρ) based on the relative abundances of the 30 most abundant genera across the eight sediment samples (S1–S8). Genera are ordered according to hierarchical clustering (average linkage) applied to the correlation matrix to highlight co-occurrence modules. Only strong correlations (|ρ| ≥ 0.7) are displayed on the color scale; weaker correlations are shown in white. Positive correlations indicate genera that tend to increase or decrease together across stations, suggesting potential co-occurrence or shared niche preferences rather than direct biotic interactions.

The co-occurrence analysis at the genus level revealed several distinct clusters of taxonomically and functionally related fungi (**Figure 5**). The most prominent module grouped the phytopathogenic genera *Ascochyta*/*Didymella* with *Penicillium*, *Talaromyces*, *Ceramothyrium*, *Calophoma*, *Lasiodiplodia*, *Fusarium*, *Gibberella*, *Xenodidymella*, *Rhizoctonia*, and an unidentified taxon. These genera are frequently associated with plant tissues as pathogens, endophytes, or saprotrophs, and their strong positive correlations (Spearman ρ ≥ 0.7) suggest that they respond similarly to inputs of plant litter and root material, and possibly to the arrival of infected plant debris from surrounding agricultural areas.

A second major module linked wood-decaying polypores, such as *Antrodia* and *Ganoderma*, with putative ectomycorrhizal or dark-septate fungi (*Acanthostigma*, *Darksidea*, *Geoglossum*) and several yeast genera (*Saccharomyces*, *Kazachstania*, *Rhodotorula*, *Malassezia*), as well as soil saprotrophs such as *Mortierella* and *Geastrum*. This assemblage likely reflects a niche associated with the degradation of more recalcitrant woody debris and microhabitats with fluctuating redox and salinity conditions, where filamentous decomposers and osmotolerant yeasts co-occur. Overall, these co-occurrence patterns indicate that sediment mycobiomes in the REMC are structured into functionally coherent assemblages rather than random aggregations of taxa, reinforcing the idea that dominant genera such as *Ascochyta*/*Didymella* may act as indicators of specific environmental states or disturbance regimes.

**Figure 6:**
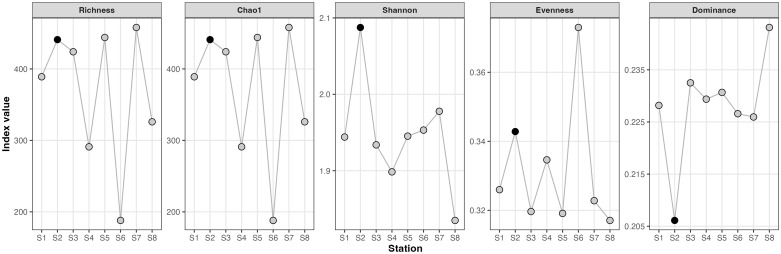
Alpha-diversity patterns of sediment fungal communities along the “La Flora” trail in the REMC. Observed richness, Chao1 estimated richness, Shannon diversity, Pielou’s evenness, and Simpson dominance are shown for each sediment station (S1–S8). Points represent individual stations connected by lines to illustrate trends along the transect; station S2 (sample 2005L2-71) is highlighted in black.

Regarding diversity, the sites 2005L2-68, 69, and 70 were the richest in this study, with species counts of 541, 553, and 574, respectively. Site 2005L2-69 was the most diverse among them, as indicated by the Shannon index (H’ = 2.166). Conversely, site 2005L2-75 exhibited the lowest diversity, with 359 species (**Figure 6**). Notably, site 2005L2-71 had the lowest species count at 204, though it was not the least diverse according to the Shannon index.

**Figure 7:**
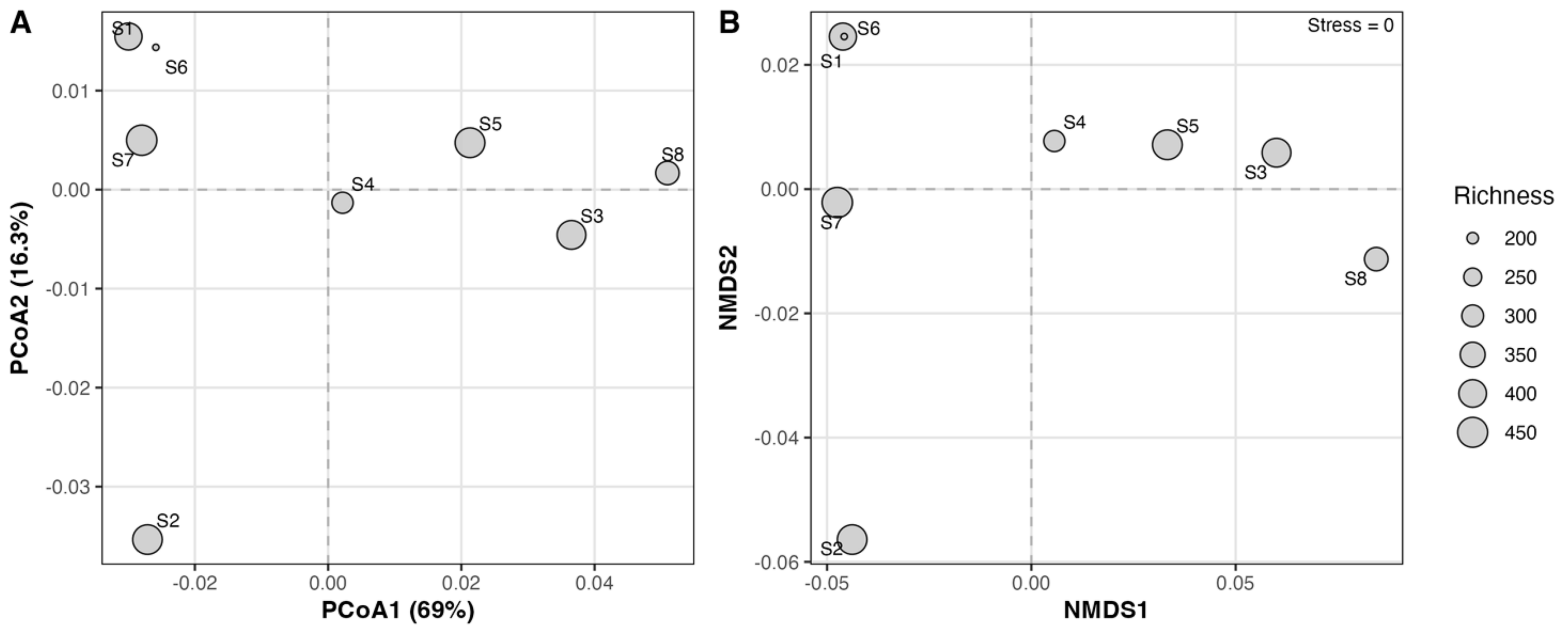
Ordination of sediment fungal communities in the REMC. **(A)** Principal Coordinate Analysis (PCoA) based on Bray–Curtis dissimilarities of genus-level relative abundances for the eight sediment stations (S1–S8). **(B)** Non-metric Multidimensional Scaling (NMDS) of the same Bray–Curtis distance matrix; the stress value of the NMDS solution is shown in the upper-right corner. In both panels, point size is proportional to genus richness at each station, dashed lines indicate the origin of each ordination axis, and sample labels (S1–S8) are displayed next to their corresponding points.

Both PCoA and NMDS ordinations indicate that fungal assemblages in the REMC sediments are overall similar among stations, forming a compact cluster in multivariate space (**Figure 7**). However, station S2 (sample 2005L2-71) is clearly displaced along the principal ordination axes, suggesting a distinct community composition compared to the remaining sites, despite comparable richness levels.

## 4 Discussion

Our metabarcoding survey provides initial molecular evidence that the sediments of Ecuador’s Reserva Ecológica Manglares Churute (REMC) harbor a diverse fungal community. This community is structurally aligned with global mangrove patterns but distinguished by a notable and unexpected abundance of potential phytopathogenic taxa. Situated within a protected yet anthropogenically influenced reserve, this finding establishes a vital baseline for understanding the relationship between fungal diversity, ecosystem function, and anthropogenic stress in a region of the Neotropics that remains critically understudied.

### 4.1 Advancing beyond morphological limitations in REMC

The findings of this study constitute a significant methodological and conceptual advancement over previous knowledge concerning fungi in the REMC. Previous research has been limited to morphological identification of a subset of cultivable, predominantly lignicolous marine fungi (Álvarez et al., 2019; Tamayo-Cevallos and Álvarez-Montero, 2022). While such methods are valuable for taxonomy, they inherently overlook the extensive unculturable component of the mycobiome, often referred to as “dark matter.” Our metabarcoding analysis not only corroborates the presence of established phyla such as Ascomycota and Basidiomycota but also uncovers a considerably greater diversity, encompassing 214 genera across 10 phyla. Additionally, it highlights the ecological dominance of sediment-dwelling genera such as *Ascochyta*, *Antrodia*, and *Talaromyces*, which were not detected in earlier morphological examinations. This transition from a substrate-specific (wood) to a comprehensive (sediment) perspective fundamentally redefines our understanding of fungal biodiversity within this reserve.

### 4.2 Regional context and site-specific novelty within Ecuador

When situated within the emerging field of molecular mycology in Ecuadorian mangroves, our research emphasizes both regional patterns and site-specific unique attributes. A recent metabarcoding survey in a wetland adjacent to a mangrove on Santay Island, focusing on the rhizosphere of *Cyperus rotundus*, identified a fungal community dominated by *Cladosporium* and *Talaromyces* (Portalanza et al., 2025). Conversely, our direct sediment analysis conducted at the REMC reveals a markedly different assemblage, primarily characterized by *Ascochyta* and *Antrodia*. This discrepancy highlights that fungal communities are not solely habitat-specific—such as wetland versus mangrove sediment—but are also highly responsive to local environmental parameters and biotic interactions. The selection of the “La Flora” trail, a site with high tourist activity within a “Strict Nature Reserve,” was made to evaluate the mycobiome at the intersection of conservation efforts and anthropogenic influence. The elevated relative abundance of *Ascochyta*, a genus that encompasses known phytopathogens, may serve as a bioindicator of ecosystem stress in this setting. This hypothesis has not been tested in less disturbed Ecuadorian environments.

In our study, *Didymella rabiei* was the most abundant fungal species across all samples, despite being typically recognized as a necrotrophic fungus responsible for *Ascochyta* blight in chickpeas (*Cicer arietinum*) (Singh et al., 2022). Its presence is unusual in mangrove ecosystems, suggesting potential dispersal facilitated by wind, water, or human activities. Furthermore, its heterothallic reproductive system, which promotes allelic recombination and the emergence of new genetic variants (Gayacharan et al., 2020; Crociara et al., 2022), may confer high adaptive potential. This could indicate ecological plasticity or an ongoing adaptive expansion into the mangrove environment. The marked abundance of *Ascochyta* across all our samples, despite the lack of measured data on environmental stressors, suggests that this taxon could serve as a bioindicator. Further research combining barcode metacoding with physicochemical analysis is needed to determine whether its predominance correlates with specific stressors (e.g., elevated nutrient and pollutant levels) associated with tourism in the “La Flora” area.

### 4.3 Global comparison and ecological interpretation of dominant taxa

Globally, the dominance of Ascomycota (68%) and Basidiomycota (30%) in REMC sediments aligns with a well-established pattern observed in mangrove sediments from Southeast Asia to the Americas (Lee et al., 2019; Wainwright et al., 2023; Pothayi et al., 2024). Inserting our REMC data into the expanding corpus of global mangrove mycobiome research uncovers both convergent patterns and noteworthy divergences. Studies conducted in geographically distant regions, such as the mangrove sediments of Kerala, India (Pothayi et al., 2024), and Guangdong, China (Wu et al., 2023), confirm the widespread predominance of Ascomycota and Basidiomycota, with Dothideomycetes frequently recognized as a dominant class. This structural consistency supports the hypothesis of a conserved core functional framework for mangrove sediment fungi, presumably driven by common environmental challenges, including anoxia, salinity, and recalcitrant carbon sources (Arfi et al., 2012; Sanka Loganathachetti et al., 2017). Nevertheless, the specific genus-level composition provides a more nuanced ecological perspective. The elevated abundance of *Antrodia* (a recognized lignocellulose degrader) and *Talaromyces* (a prolific producer of hydrolytic enzymes and secondary metabolites) suggests a community actively engaged in processing the recalcitrant organic matter prevalent in mangrove sediments (Spirin et al., 2013; Nicoletti et al., 2018). This functional profile aligns with findings from mangroves in China and India, where similar saprotrophic and wood-decay guilds are predominant.

Our metabarcoding survey of Ecuador’s Reserva Ecológica Manglares Churute (REMC) reveals a fungal community dominated by Ascomycota (68%) and Basidiomycota (30%), with notable abundances of *Ascochyta*, *Antrodia*, and *Talaromyces*. In contrast, Gaafar et al. (2025) employed traditional isolation and molecular identification methods to study mangrove fungi in Saudi Arabia’s Tarout Island, reporting 17 species—primarily Ascomycota—with *Ascocylindrica marina* as the predominant taxon (30.07%). Methodologically, our approach captured a broader, unculturable diversity, including potential phytopathogens and saprotrophs. Conversely, Gaafar et al. focused on cultivable, wood-decaying fungi, providing detailed morphological and phylogenetic insights into key halotolerant species. Ecologically, both studies underscore the influence of environmental filters: Gaafar et al. identified salinity and nutrients as primary drivers, with *A. marina* abundance strongly correlated with high salinity. Although our study lacked concurrent physicochemical data, the prevalence of *Ascochyta*—a genus not reported by Gaafar et al.—may reflect site-specific stressors such as anthropogenic impact or nutrient availability. These differences highlight how methodological choices (metabarcoding versus isolation) and regional environmental conditions shape the observed fungal communities, thereby offering complementary perspectives to the global understanding of mangrove fungal ecology.

The most striking and potentially significant finding is the dominance of *Ascochyta*-related reads (exemplified by *Didymella rabiei*), which accounted for over 26% of the community. While *Ascochyta* spp. are canonical phytopathogens of terrestrial crops like chickpea (Singh et al., 2022), their ecology in mangrove systems remains poorly understood. Nonetheless, the conspicuous dominance of the phytopathogen-associated genus *Ascochyta* observed in our study is not commonly documented in these Asian systems, where communities frequently emphasize saprotrophic genera such as *Aspergillus*, *Penicillium*, or *Cladosporium* (Wu et al., 2023; Pothayi et al., 2024). This divergence underscores the influence of local biotic and anthropogenic factors that may override broad biogeographic patterns. Furthermore, our findings are consistent with those reported by Zhang et al. (2021), who documented a high diversity of basal fungal lineages in Chinese mangroves and emphasized the significant contribution of stochastic processes to community assembly, particularly in heterogeneous sediment environments. This pattern is not commonly reported as dominant in other mangrove metabarcoding studies, which more frequently highlight saprophytic or endophytic dominants (e.g., *Cladosporium*, *Penicillium*, *Trichoderma*) (Chi et al., 2019; Lee et al., 2019). Its prominence in the REMC could be interpreted through several, non-exclusive lenses: (1) as a cryptic saprotroph or endophyte exploiting mangrove-derived organic matter, (2) as a stress-tolerant taxon proliferating under the specific anthropogenic pressures of the “La Flora” trail, or (3) as an indicator of pathogenic pressure on the mangrove vegetation itself. The strong co-occurrence of *Ascochyta phacae* with saprotrophs like *Penicillium corylophilum* (**Figure 5**) may support the first hypothesis, suggesting a shared niche in processing similar organic substrates. This finding warrants targeted cultivation and pathogenicity assays to determine its proper ecological role and potential threat.

### 4.4 Insights derived from co-occurrence and diversity patterns

The co-occurrence network depicted in **Figure 5** demonstrated ecologically plausible associations, such as the clustering of lignin-degrading genera (*Antrodia*, *Ganoderma*) and the linkage of *Ascochyta* with fungi involved in metabolite production. These patterns suggest the existence of potential functional guilds or shared responses to unmeasured environmental gradients, including salinity, redox potential, or leaf litter quality. While we recognize the absence of concurrent physicochemical data as a limitation—a common challenge in preliminary baseline studies—these hypothesized associations offer a valuable framework for future hypothesis-driven research. Such research could integrate metabarcoding techniques with environmental metrology.

Indices of alpha diversity and ordination analyses indicate a predominantly homogeneous fungal community throughout the sampled transect, with a singular notable outlier at site 2005L2-71. This location, characterized by the lowest richness yet highest evenness, was distinctly segregated in PCoA/NMDS plots. In the absence of environmental data, it is speculative to attribute this to localized factors such as microtopography, root density, or historical disturbances that may have generated a unique niche. This spatial heterogeneity, even at small scales, mirrors findings from other mangrove ecosystems where sediment depth and microenvironmental conditions significantly influence fungal community structures (Zhang et al., 2021; Ng et al., 2025). The rarefaction curves substantiate that our sampling efforts captured a considerable fraction of the local biodiversity; nonetheless, the complete fungal richness of the REMC remains to be fully elucidated.

### 4.5 Biotechnological implications and conservation outlook

The detection of genera with well-documented biotechnological potential, such as *Talaromyces* and *Penicillium* (sources of novel antimicrobials and enzymes), and *Antrodia* (a producer of bioactive compounds), underscores the REMC’s value as a reservoir for biodiscovery (He et al., 2019; Wei et al., 2021). Our study advances beyond traditional cultivation-based bioprospecting by providing a DNA-based inventory that prioritizes these taxa for future isolation efforts. Methodologically, this work affirms the importance of metabarcoding in mangrove mycology, as it captures the “microbial dark matter” that would otherwise escape cultivation-based surveys (Li S. et al., 2023).

Our study thus bridges a critical gap between ecological discovery and applied microbiology, offering a roadmap for biodiscovery in Neotropical mangroves, a priority highlighted by the Mangrove Microbiome Initiative (Allard et al., 2020). Methodologically, this work demonstrates the necessity of DNA-based metabarcoding approaches over culturing techniques, as 15–30% of the detected taxa belong to the microbial “dark matter” (Li M. et al., 2023). Traditional methods would have missed these lineages, distorting diversity estimates. The rarefaction curves (**Figure 4**) further confirm that even high-throughput sequencing underestimates true fungal richness, underscoring the need for larger-scale sampling in future studies.

## 5 Conclusions

In conclusion, this study establishes the first molecular baseline for fungal diversity in the REMC, thereby filling a critical gap in Neotropical mangrove microbiome research. By revealing a community that is globally familiar in its structure yet uniquely characterized by a potential phytopathogen dominating, we emphasize the complex interplay between conserved ecological functions and site-specific disturbances. Future research should build upon this baseline by: (1) integrating comprehensive environmental metadata to correlate community composition with drivers such as salinity, nutrients, and pollutants; (2) employing spatial and temporal replicated sampling to assess the stability and drivers of the *Ascochyta* signal thoroughly; and (3) initiating cultivation campaigns to isolate taxa of ecological and biotechnological interest identified herein. As mangrove ecosystems confront escalating threats, understanding their microbiome—including potential pathogens and beneficial decomposers—is not merely an academic pursuit but a crucial component of informed conservation, restoration, and sustainable management strategies. This study fills a crucial gap in Neotropical mangrove microbiology and highlights the power of metabarcoding analysis to reveal microbial “dark matter” that traditional methods overlook. As mangroves face increasing threats from climate change and human activities, understanding their microbial allies and enemies is vital for conservation and restoration efforts. The results support incorporating microbiome monitoring into mangrove management to help these ecosystems protect coastlines, store carbon, and maintain biodiversity in a changing world.

## Call to action

Future research must expand sampling across spatiotemporal scales to decode fungal functional roles and harness their potential for ecological and biotechnological innovation. Protecting mangroves means protecting their microbiome—a silent guardian of global ecosystem health.

## Data Availability

The original contributions presented in the study are publicly available. This data can be found here: NCBI BioProject PRJNA1413243. BioSamples accessions: SAMN54872965, SAMN54872966, SAMN54872967, SAMN54872968, SAMN54872969, SAMN54872970, SAMN54872971, SAMN54872972.

## Supplementary material

Supplementary Table 1 Relative abundance (%) of the five most dominant fungal genera in each mangrove sediment sample.
