# Supplementary material for: Metabarcoding insights into the fungal diversity and biotechnological potential of mangrove sediments in Ecuador’s Reserva Ecológica Manglares Churute
Source: Front Fungal Biol. 2026 Feb 10;7:1710970. doi: 10.3389/ffunb.2026.1710970 (PMC12929412; doi:10.3389/ffunb.2026.1710970)
Supplement: Supplementary Table 1 — Relative abundance (%) of the five most dominant fungal genera in each mangrove sediment sample. [file Table1.docx]

**Supplementary Material**

**Table S1.** Relative abundance (%) of the five most dominant fungal genera in each mangrove sediment sample.

|  | **Sediment Samples** | | | | | | | |
| --- | --- | --- | --- | --- | --- | --- | --- | --- |
|  | **2005L2_68** | **2005L2_69** | **2005L2_70** | **2005L2_71** | **2005L2_72** | **2005L2_73** | **2005L2_74** | **2005L2_75** |
| *Ascochyta* | 28.54 | 25.09 | 26.37 | 27.21 | 27.05 | 28.49 | 29.04 | 26.75 |
| *Antrodia* | 21.07 | 21.79 | 25.92 | 23.24 | 24.76 | 21.38 | 21.81 | 28.01 |
| *Talaromyces* | 18.22 | 17.64 | 16.19 | 17.3 | 16.56 | 17.33 | 17.53 | 15.75 |
| *Penicillium* | 5.65 | 5.93 | 4.93 | 5.29 | 5.18 | 5.76 | 5.64 | 4.79 |
| *Ceramothyrium* | 1.35 | 1.63 | 1.36 | 1.38 | 1.41 | 1.26 | 1.52 | 1.39 |
| *Rhizoctonia* | 0.94 | 1.55 | 0.97 | 1.09 | 0.96 | 0.98 | 1.00 | 1.09 |
